# Coronavirus Disease-Associated Mucormycosis from a Tertiary Care Hospital in India: A Case Series

**DOI:** 10.7759/cureus.16152

**Published:** 2021-07-03

**Authors:** Yudhyavir Singh, Venkata Ganesh, Shailendra Kumar, Nishant Patel, Richa Aggarwala, Kapil Dev Soni, Anjan Trikha

**Affiliations:** 1 Anaesthesiology, Critical Care and Pain Medicine, All India Institute of Medical Sciences, New Delhi, New Delhi, IND; 2 Critical and Intensive Care, Jai Prakash Narayan Apex Trauma Centre, All India Institute of Medical Sciences, New Delhi, New Delhi, IND

**Keywords:** mucormycosis, covid-19, steroids, cam, diabetes mellitus

## Abstract

Background: Coronavirus disease (COVID-19) remains a health concern with new challenges emerging as the pandemic progresses. The recent rise of opportunistic infections especially mucormycosis in COVID-19 patients is further complicating their outcomes. Mucormycosis is well known to infect patients with diabetes mellitus, malignancy, chemotherapy, and other immunocompromised conditions. The treatment of COVID-19 largely remains systemic steroids and other immunomodulators that add to the risk of invasive fungal infection.

Methodology: Here, we present a retrospective case series of 13 patients with individual clinical characteristics along with the demography and treatment details. The data were collected retrospectively in a single center that caters to a large population of COVID-19 patients with varying severity.

Results: Thirteen patients were presented with COVID-19 associated mucormycosis (CAM). The median age was higher in non-survivors (49.5 years), with a higher odds of death (23.8) in those with severe COVID, having overall mortality of 64.3%. Moreover, diabetes mellitus was present in 61.5% of patients with a mortality of 75%. About 11 (84.6%) patients had received prior steroids for COVID-19. The incidence of hyperglycemia at admission was equal among both survivors and non-survivors.

Conclusion: The prevalence of mucormycosis seems to be increasing among COVID-19 patients which may be associated with increased use of steroids, the possible immunocompromised state imposed by SARS-CoV-2, or co-existing conditions such as diabetes mellitus. The mortality of CAM is remarkably high and apart from preventive practices and rational use of immunomodulators, a high index of suspicion with early diagnosis would be key to survival.

## Introduction

Coronavirus disease 2019 (COVID-19) pandemic continues to be a major health problem worldwide. The presentation of COVID-19 has been diverse, ranging from mild (flu-like symptoms) to severe life-threatening pneumonia with multiorgan involvement [1]. Despite being more than one year into this pandemic, the definitive treatment of COVID-19 continuous to be controversial. However, systemic steroids have shown some survival benefits. On the flip side, rampant use of glucocorticoids has potentially resulted in secondary bacterial and fungal infections [2]. This increased incidence of secondary infections may also be attributed to preexisting morbidities such as poorly controlled diabetes mellitus, lung diseases, malignancy, and the immunocompromised state. In the early part of the pandemic, less than 1% of secondary infections were fungal [3]. Usually, bacterial infections are the most common cause of secondary infection in COVID-19 but recent reports of a rise in systemic fungal infections, particularly invasive mold, in India have raised a lot of concern.

In the general population, in the pre-COVID era, the incidence of mucormycosis is very low, varying from 0.005 to 1.7 per million [4]. In the 2003 outbreak of SARS CoV infection, the incidence of fungal infection was 14.8-27%, and it was the main cause of death for severe acute respiratory syndrome patients, accounting for 25-73.7% of all causes of death [5-7]. The lesson learned from 2003 shows that there was a high probability of increased incidence of fungal infection in SARS CoV affected or recovered patients, like the finding being seen now with SARS-CoV-2. Here, we present a case series of 13 patients having COVID-19 infection along with mucormycosis needing hospitalization.

## Materials and methods

Case presentation

Since the outbreak of coronavirus in India, our dedicated COVID hospital has cared for thousands of patients. Of these, we found 13 patients with mucormycosis between November 2020 to January 2021. For this case series, the relevant data were collected retrospectively. The patient with the diagnosis of mucormycosis was identified with a positive KOH mount and associated clinical features suggestive of fungal infection.

Demographic details including age and gender, clinical and laboratory data along with the comorbidities and duration of hospital stay were noted. Details pertaining to the mode of respiratory support given, COVID severity, steroid therapy, antifungal therapy, and any surgical intervention required were also collected (Table 1). Moreover, the outcome (death/discharge) of the patients noted.

**Table 1:** Individual patients characteristics ALL: acute lymphoblastic leukaemia, Ca: cancer, CAD: coronary artery disease, CLD: chronic liver disease, DKA: diabetic ketoacidosis, DM: diabetes mellitus, HE: hepatic encephalopathy, HTN: hypertension, IPPV: invasive positive pressure ventilation, NHL: non-Hodgkin lymphoma, PTCA: percutaneous coronary angiography, TB: tuberculosis.

Age in years	Gender	Comorbidities	Time of diagnosing mucormycosis	Type of mucormycosis	Symptoms at presentation	COVID Severity	Maximum respiratory support	Surgical intervention	Steroid	Hospital stay in days	Outcome
38	Male	HTN, post-renal transplant	In-hospital, 7^th^ day of admission	Rhino orbital	Fever, cough, shortness of breath, epistaxis	Severe	IPPV	No	Yes	29	Death
50	Female	HTN, DM, DKA, hypothyroid	Previously diagnosed	Rhino orbital	Facial swelling, epistaxis	Severe	IPPV	No	Yes	5	Death
55	Male	HTN, DM, bronchial asthma	Previously diagnosed	Rhino orbital	Pain, swelling, redness left eye, headache	Severe	IPPV	No	Yes	7	Death
46	Female	Ca breast, DM, HTN	In-hospital, 16^th^ day of admission	Orbital and PNS	Fever, cough, shortness of breath	Severe	IPPV	No	Yes	23	Death
38	Male	DM, CAD, post-PTCA	Previously diagnosed	Right sino-orbital	Swelling of the right eye and cheek	Mild	Room air	No	No	24	Discharge
75	Male	DM, HTN, CAD	Previously diagnosed	Invasive Rhino-orbital Mucormycosis with palatal perforation	Pain and swelling of face	Severe	IPPV	No	Yes	12	Death
28	Male	None	Previously diagnosed	Rhino-orbital	Swelling and watering from eyes, loss of vision	Mild	Room air	Yes	No	17	Discharge
32	Male	Seizure	Previously diagnosed	Orbital	Swelling of face, redness, and protrusion of the eye	Mild	Face mask	Yes	No	12	Discharge
49	Male	DM, CLD, HE	Previously diagnosed	Rhino-cerebral	Fever, cough, shortness of breath	Severe	IPPV	No	Yes	7	Death
37	Female	HTN, DM	Previously diagnosed	Rhino-orbital	Swelling of face	Severe	IPPV	No	Yes	22	DOR
59	Male	HTN, DM, NHL, hypothyroid	Previously diagnosed	Paranasal sinus mucormycosis	Shortness of breath, nasal bleeding	Severe	IPPV	Yes	Yes	2	Death
5	Male	B cell ALL	Previously diagnosed	Paranasal sinus and cerebral mucormycosis	Loose motion	Severe	IPPV	No	Yes	8	Death
26	Male	Febrile neutropenia, B cell ALL	Previously diagnosed	Rhino-orbital	Fever, cough, Shortness of breath	Moderate	Face mask	No	Yes	22	Discharge
30	Male	Disseminated TB	Previously diagnosed	Pulmonary	Fever, cough, shortness of breath, lower limb weakness, altered sensorium	Severe	IPPV	No	Yes	8	Death

In statistical analysis, the data were described on either continuous or categorical variables. The continuous variables were presented as mean with standard deviation or median with interquartile range, while the categorical variables were presented as frequency and percentages. The chi-square test, student t-test, and Mann-Whitney test were applied as appropriate. The downside when it comes to the generalizability of findings is that the sample size is small.

## Results

**Figure 1:**
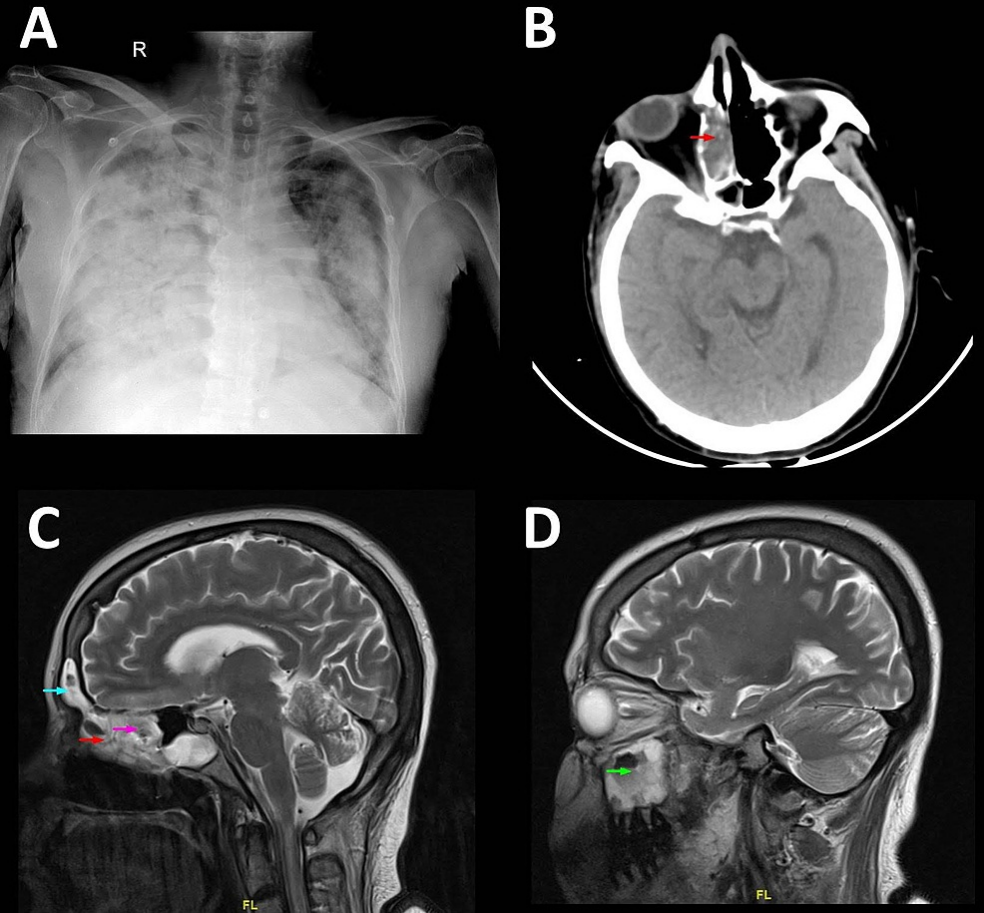
Representative images (A) Chest X-ray of a patient with mucormycosis; (B) axial non-contrast computed tomography of head showing right ethmoid sinusitis (red arrow); (C) T2 MRI head sagittal section showing right ethmoid sinusitis (red arrow), sphenoid sinusitis (magenta arrow) and right frontal sinusitis (blue arrow); (D) maxillary sinusitis (green arrow); the sinusitis is visualized as increased mucosal thickening suggesting fungal infection.

A total of 13 patients admitted with RTPCR positive of COVID-19 diagnosed with mucormycosis over three months. Out of 13, eight patients had rhino-orbital involvement, two patients had rhino-cerebral involvement, two had only paranasal sinus involvement and lastly one (n=1) had pulmonary involvement (representative imaging findings are in Figure 1). Only two patients were diagnosed with symptoms of rhino-orbital mucormycosis during the treatment at our hospital (7-16 days); the rest all were diagnosed before admission and admitted with symptoms of mucormycosis. For description, we divided the cohort into survivors and non-survivors and demographic and clinical data presented in Table 2.

**Table 2:** Demographic and clinical data Median (IQR) or n (%) have been presented where appropriate. Column percentages are in parentheses of n (%). *p-value < 0.05 has been statistically significant. Mann-Whitney U test has been used for numerical data and Fischer’s exact for categorical data. ^‡^The purely pulmonary mucormycosis case was excluded in this odds ratio calculation. DM: diabetes mellitus; HTN: hypertension; CAD: coronary artery disease; CLD: chronic liver disease; CKD: chronic kidney disease; CCI: Charlson comorbidity index; TLC: total leucocyte count; PLT: platelet count; CRP: C-reactive protein; IL6: interleukin-6; SGOT: serum glutamic-oxaloacetic transaminase; SGPT: serum glutamic pyruvic transaminase; Na: serum sodium; K: serum potassium; RBS: random blood sugar at admission; LFT: liver function test; KFT: kidney function test; L-AMB: liposomal amphotericin B.

Variable	Full cohort (n=13)	Survivors (n=5)	Non-survivors (n=8)	P- value	Odds ratio for mortality (95% CI)
Age (years)	38 (31–52)	32 (27–37.5)	49.5 (40–58)	0.011*	
Gender					
Female	3 (23.1%)	1 (20%)	2 (25%)	1	1.33 (0.88 - 20.10) odd of death female to male
Male	10 (76.9%)	4 (80%)	6 (75%)		
Comorbidities					
DM	8 (61.5%)	2 (40%)	6 (75%)	0.293	
HTN	7 (53.8%)	1 (20%)	6 (75%)	0.103	
CAD	2 (15.4%)	1 (20%)	1 (12.5%)	1	
CLD	1 (7.7%)	0 (0%)	1 (12.5%)	1	
CKD	0	0	0	-	
Malignancy	3 (23.1%)	1 (20%)	2 (25%)	1	
Asthma	1 (7.7%)	0 (0)	1 (12.5%)	1	
CCI	3 (0–5)	2 (0–2.5)	4.5 (0.75–5.75)	0.93	
COVID severity					
Severe	10 (76.9%)	2 (40%)	8 (100%)	0.035*	23.8 (0.89–633.56)*
Non-severe	3 (23.1%)	3 (60%)	0 (0%)		
ICU admission	10 (76.9%)	2 (40%)	8 (100%)	0.035*	
Hospital-stay (days)	12 (7–22.5)	22 (14.5–23)	7.5 (5.5–20.25)	0.127	
Surgical outcome	2 (15.4%)	1 (12.5%)	1 (20%)	1	1.5 (0.07 – 31.57)^ ‡^ odds of death when not operated
Mortality	8 (61.53%)	-	-	-	-
Laboratory parameters (baseline)					
Haemoglobin	9.5 (7.5–11.5)	11.1 (7.5–14.4)	8.2 (7.52–10.87)	0.284	
TLC	7800 (6250–14050)	14100 (6755–15900)	7675 (5875–12075)	0.171	
PLT	197,000 (104,000–276,000)	213,000 (115,500–329,500)	166,000 (79,000–226,000)	0.524	
CRP	15.58 (7.95–23.2)	13.63 (12.19–13.63)	17.13 (5.37–26.48)	0.643	
IL6	483.23 (47.65–1308.87)	61.27 (34.0–61.27)	1063.64 (75.03–1399.56)	0.286	
Ferritin	920.1 (426.2 – 1373)	801.80 (611.6 -801.80)	920.10 (361.72 -1500)	1	
Bilirubin	0.9 (0.6–1)	1.0 (0.75– 1.05)	0.70 (0.52–0.9)	0.171	
Albumin	2.4 (2.15–2.55)	2.5 (1.85–2.5)	2.30 (2.13–2.9)	1	
SGOT	37 (27–26.5)	34 (19–45.5)	51 (30.25–60.25)	0.22	
SGPT	34 (24.5–54)	36 (28.5–54.5)	32 (23.75–50.25)	0.524	
Urea	38 (24–63)	32 (23–125)	43.5 (25.25–72)	0.524	
Creatinine	0.9 (0.6– 1.4)	0.9 (0.7–4.4)	0.95 (0.53–1.33)	0.622	
Na	135 (132–143)	135 (129–137.5)	135 (132–147.75)	0.524	
K	4.3 (4.45–4.8)	4.3 (2.95–4.65)	4.4 (3.7–4.88)	0.524	
RBS	220 (200–280)	280 (180–280)	220 (200–255)	0.882	
Laboratory parameters (course in hospital)					
LFT derangement	4 (30.8%)	2 (40%)	2 (25%)	1	0.5 (0.45–5.51)
RFT derangement	11 (84.6%)	4 (80%)	7 (87.5%)	1	1.75 (0.84–36.28)
Dyselectrolytemia					
Hypokalaemia	9 (69.2%)	5 (100%)	4 (50%)	0.105	11 (0.45–263.54)
Hyperkalaemia	4 (30.8%)	0 (0%)	4 (50%)		
Treatment received					
Antifungal therapy					
a. L-AMB (only)	11 (84.6%)	4 (80%)	7 (87.5%)	1	1.75 (0.84–36.28) odds of death when receiving only L-AMB
b. Posaconazole (additional)	2 (15.4%)	1 (20%)	1 (12.5%)		
Remdesivir	2 (15.4%)	0 (0%)	2 (25%)	0.487	4.23 (0.164–108.22)
Tocilizumab	1	0 (0%)	1 (20%)	0.385	0.17 (0.005–5.27)

**Figure 2:**
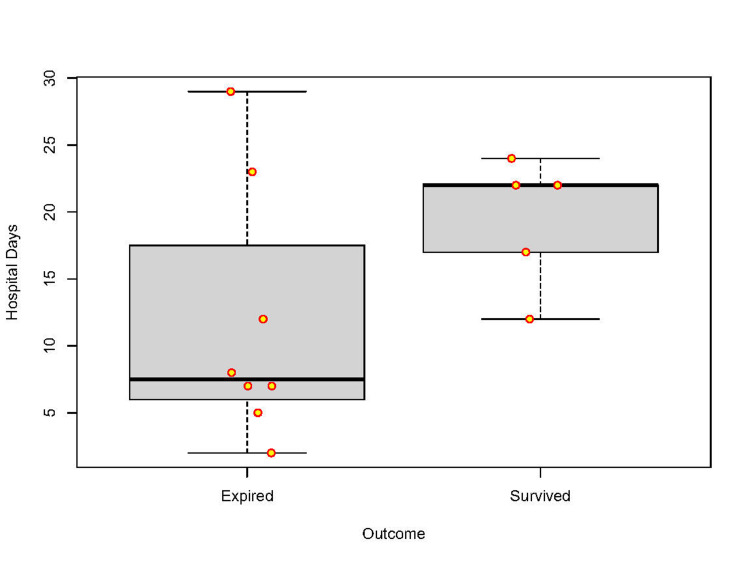
Boxplot of hospital stay in days among non-survivors and survivors

We noticed that the non-survivors had a higher median age (median age of 49.5 vs 32.5, p-value of 0.011). All the non-survivors had severe COVID and the need for higher FiO_2_, HFNC, or intubation at admission. Those categorised as severe COVID had an odd of death of 23.8 [(0.89-633.56), p < 0.05]. The odds of death were 28.6 [(1.118-731.58), p < 0.05] when the patient needed invasive ventilation on admission. All the non-survivors needed invasive ventilation during their hospital stay. The overall mortality was 64.3% (9/14 patients including the child). Excluding the cases of purely pulmonary mucormycosis as well as the child, the odds of death when not operated on was 1.5 [(0.07-31.57), p > 0.05]. Both the survivors and non-survivors experienced equivalent rates of electrolyte abnormalities and worsening in the liver and renal function tests. There was no significant difference in the incidence of renal injury at admission defined in terms of serum creatinine and oliguria or in the incidence of leukopenia or leucocytosis between survivors and non-survivors at the time of admission. The positivity rate for KOH mount (biopsy or sputum) was equally distributed between survivors and non-survivors. All the patients received methylprednisolone because of co-existing COVID pneumonia. The numbers presented are purely for descriptive reasons and the generalizability of these findings is limited considering the small sample size. The median hospital stay was 7.5 (5.5-20.25) among those who expired and 22 (14.5-23) among survivors (Figure 2). Among the surviving five patients, the only additional risk factor for getting a fungal infection that was identifiable was that two of them had received recent chemotherapy.

## Discussion

A year into the pandemic, and yet the only reasonably effective treatment options we have for COVID-19 are oxygen therapy and immunosuppressants such as steroids and tocilizumab [8,9]. However, the use of steroids is known to raise the risk of secondary infection [10]. Until recently, few case reports have been published, but a firm, potentially catalytic association between COVID-19 and increased fungal infections still needs to be verified. Maini et al. reported a sole case of a 38-year-old male with rhino-orbital mucormycosis in COVID-19 in May 2021 [11]. A two-case report of mucormycosis was also published in April 2021, where both the patients had received corticosteroids therapy during COVID-19 [12]. Similar case reports have been published by Werthman-Ehrenreich [4], Mehta and Pandey [12], and Revannavar et al. [13] on rhino-orbital mucormycosis.

In our study, a total of 14 cases were reported, of which one was a pediatric patient. There was a male predominance in our study population 11 (78.5%) which is similar to previous studies [2,3,7]. This child was excluded from our analysis. The overall median age was 38 (31-52) years, of which the survivors had a median age of 32 (27-37.5) years and in non-survivors the median age was 49.5 (40-58) years, indicating high mortality in the older age group. Moreover, overall mortality (61.5%, 8 out of 13 patients were non-survivors) was also high. Like our results, Patel et al. also found an increased risk of death in age >54 years in their study [14]. Recently, Garg et al. in their systematic review on Coronavirus-associated mucormycosis (CAM) reported seven deaths (87.5%) and only one survival pointing towards high mortality due to mucormycosis with the median age of patients found to be 57.5 (22-86 years) similar to our study [15].

Diabetes mellitus has been associated with severity in COVID-19. It has been seen that diabetics are at increased risk of dying than those without this and a high mortality rate with CAM remains the main concern [16,17]. In our research, diabetes mellitus (n=8, 61.5%) was the most common predisposing factor and mortality was higher in diabetic patients (n=6, 75%); none of the cases was diagnosed diabetes on admission. Additionally, we found high median values of random blood sugar on admission, indicating poor control of diabetes but we could not obtain glycated hemoglobin values. Similar findings were reported in the study by Garg et al. about diabetes mellitus [15]. Moreover, Sharma et al. in his prospective observational case series of 23 mucormycosis patients reported diabetes mellitus in the majority of the patients (n=21, 91.3%) with uncontrolled diabetes in 12 of them [18]. Also, in a multicentre, retrospective study, Patel et al. concluded that the most common underlying disease among both CAM and non-CAM groups was uncontrolled diabetes (62.7%) [14]. SARS-CoV-2 has been shown to affect the beta cells of the pancreas, causing metabolic derangement potentially leading to diabetes mellitus [19]. From the above facts, it is difficult to determine if the highly prevalent SARS-CoV-2 causes a diabetes-like environment in the population predisposing them to mucormycosis or if this increased prevalence of mucormycosis happening due to increased home-based anti-COVID steroid therapy.

Furthermore, poorly controlled diabetics may have underlying renal dysfunction, adding to the risk factors for fungal infections [16]. Our research data showed that most of the subjects (n=11, 84.6%) had renal dysfunction, where non-survivors (n=7, 87.5%) have more renal derangement than survivors, increasing the odds ratio of dying to 1.75. The presence of multiple risk factors and comorbidities in patients with COVID-19 along with the added immunosuppression caused by steroids and potentially other immunomodulatory therapies increases the predisposition to and the severity of fungal infection [9,10].

Steroids especially glucocorticoids have been advocated in the management of COVID-19 [20]. Most of the patients in our study (n =11, 84.6%) had received steroids for COVID -19 treatment, some even before their admission to our hospital. Only one patient had received tocilizumab as a second immunomodulator. A multicentric study found that inappropriate use of steroids was independently associated with late CAM. Similar to the original SARS-CoV, it is possible that SARS-CoV-2 also causes some immune dysregulation that predisposes the patient to invasive fungal infection, but this remains to be proven [21,22]. Therefore, from our limited case series, it is difficult to attribute causality to steroids for the development of mucormycosis. Tocilizumab use in COVID-19 has also been reported to be a risk factor for invasive fungal infection [23]. Here, in our study, only one patient had received tocilizumab in-hospital when he was already diagnosed with mucormycosis, and this was done in a compassionate sense since his oxygen requirement was going up despite all measures.

One of the causes of high mortality in CAM was also the severity of COVID-19 [1]. In our assessment, ten patients (n=10, 76.9%) of mucormycosis presented with severe COVID-19, out of which 8 (80%) were non-survivors, making the odds of death 23 times higher. Garg et al. also reported similar findings with high mortality of 87.5%, among patients with severe COVID-19 [15]. In a letter to the editor of 10 cases of orbital mucormycosis, Sarkar et al. reported a mortality of 40% [24]. Multiple case reports were published and most of the survival was poor [24,25]. Quite surprisingly in a case series of 23 patients of CAM by Sharma et al., no death was reported [18]. Multiple reasons can be for no mortality such as early diagnosis, timely starting of antifungal therapy, early debridement, and surgical intervention. Appropriate and timely antifungal therapy has been key for the management of mucormycosis. Liposomal amphotericin B is the drug of choice but other drugs such as posaconoazole and isavuconazole can also add to the management. But the evidence for combination antifungal therapy in the management of mucormycosis is not robust [26]. In addition, the timely surgical intervention also forms the key to management, if it has spread and not responding to the treatment. In our series, only three (out of 13) patients underwent surgical intervention, of whom two were discharged and the other one died. Sharma et al. in his case series reported all the 23 patients underwent surgical debridement with all of them surviving, although long-term survival and recurrence are still debatable.

Mucormycosis involving other organs system such as pulmonary, gastrointestinal, and others have also been noted in COVID-19 patients [27]. One patient diagnosed with pulmonary mucormycosis in our series was also receiving treatment for tuberculosis, also succumbed to his illness. We found two case reports with one patient in each, diagnosed with pulmonary mucormycosis (Mekki et al. and Passer et al.), with none of them surviving [28,29]. An interesting observation is that the number of pulmonary mucormycosis cases reported is less in comparison to other sites. Further several pulmonary mucormycosis cases might have not been even diagnosed due to challenges faced in obtaining the pulmonary secretions in COVID-19 patients. Besides this, one case report of gastrointestinal mucormycosis in a COVID-19 patient was found [30].

Control of hyperglycemia, prompt treatment with liposomal amphotericin B, and surgery are essential for the successful management of mucormycosis. However, COVID-19 has led to an atypical scenario. With irrational use of steroids in COVID-19 patients, hyperglycemia has been severely aggravated with a parallel increase in fungal infections. Coexisting COVID-19 severity and multiorgan involvement have added to the mortality of the patients. The high caseload of COVID-19, very high mortality in hospitalized (and intensive care unit) patients, a low index of suspicion for fungal infection, the non-specific nature of early symptoms associated with a fungal infection, in combination with an overwhelmed, understaffed laboratory serve as impediments in providing timely diagnosis and treatment to this population.

The route of the spread of mucormycosis is mainly through inhaled fungal spores [14]. The practice of wearing masks to prevent COVID-19 may equally decrease the spread of mucormycosis. Hence, high-risk populations such as diabetics and the immunocompromised should be encouraged to continue wearing masks at least till the prevalence of COVID-19 and mucormycosis decreases. Patients admitted in-hospital with steroid therapy should also be encouraged to wear a face mask, over their nasal prongs on an oxygen mask to prevent hospital-acquired mucormycosis. Two of our patients were diagnosed with hospital-acquired mucormycosis with a median time of diagnosis 7-16 days from admission. 

Few limitations we acknowledge in this case series on CAM. The first limitation is that our study summarizes data from a single-centre tertiary care hospital where most patients are referrals. This gives rise to selection bias, with complicated or partially treated patients coming to our centre for further management. The sample size is small, and the data have been collected retrospectively. Therefore, there are inaccuracies concerning the timing of starting and duration of treatment with antifungal treatment, glycated hemoglobin, previous blood sugar control, and duration of steroid received, which could not be analyzed.

## Conclusions

To sum up, clinicians must be aware of the risk of mucormycosis in patients suffering as well as recovering from COVID-19 especially those with inappropriate steroid therapy and with uncontrolled diabetes mellitus. A high index of suspicion should be there while managing COVID-19 patients as mucormycosis is potentially treatable if diagnosed early, unlike COVID-19 which has only anti-inflammatory therapies available as of now. Also, there is a need to stress the sensible use of steroids. Where there is no oxygen requirement and no evidence to suggest a florid inflammatory response, it would be prudent to avoid steroid and immunosuppressive use for COVID-19. Early and overzealous antibacterial use may also be harmful. Key to the management of mucormycosis remains early diagnosis and starting of appropriate antifungal treatment, and if required timely surgical intervention.
